# Identification of a New Peritrophic Membrane Protein from Larval *Holotrichia parallela* (Coleoptera: Motschulsky)

**DOI:** 10.3390/molecules191117799

**Published:** 2014-11-03

**Authors:** Dan Zhao, Wei Guo, Shaoya Li, Ruijun Li, Daqing Xu, Xiujun Lu

**Affiliations:** 1 Plant Science and Technology College, Beijing University of Agriculture, Beijing 102206, China; E-Mail: zhaodan198411@163.com; 2 College of Plant Protection, Agricultural University of Hebei, Baoding 071001, China; E-Mails: lshy0826@163.com (S.L.); liruijun99@sina.com (R.L.); luxiujun@hebau.edu.cn (X.L.); 3 College of Life Sciences, Agricultural University of Hebei, Baoding 071001, China; E-Mail: daqingxu2013@126.com

**Keywords:** peritrophic membrane, *Holotrichia parallela*, midgut, chitin binding protein, HpCBP45

## Abstract

Peritrophic membranes (PMs) are composed of proteins, proteoglycans and chitin that play important roles in the structural formation and function of the PM. This study identified and characterized a new chitin binding protein named HpCBP45 by immunoscreening of the *Holotrichia parallela* larvae midgut expression library. The predicted amino acid sequence indicates that it contains eight tandem chitin binding domains belonging to the peritrophin-A family. The HpCBP45 protein was expressed as a recombinant protein in the yeast *Pichia pastoris* and chitin binding assay demonstrated that recombinant HpCBP45 protein could strongly bind to chitin. qRT-PCR analysis showed that HpCBP45 was mainly localized in the midgut, further confirming the *H. parallela* PM belongs to Type I PM. The discovery and characterization of the peritrophic membrane protein HpCBP45 provides a basis for the further investigation of its biochemical and physiological functions in *H. parallela*.

## 1. Introduction

In many insects, the midgut epithelium is lined with a semi-permeable structure called the peritrophic membrane (PM) or peritrophic matrix, and the functions of this semi-permeable membrane are crucial for the protection of the midgut epithelium from mechanical damage by food particles, bacterial damage and parasite invasion [1]. The PM may also protect phytophagous insects from toxic phenolic compounds found in plants [2]. There are two types of insect PMs. Type I PM, which is mainly found in the larvae of Lepidoptera such as *Helicoverpa armigera*, *Trichoplusis ni*, *Spodoptera exigua* and has also been found in Coleoptera, Diptera, and so on, is synthesized from the entire midgut epithelium [3,4,5,6,7,8,9]. Type II PM is synthesized as a continuous sleeve from the cell of the cardia in the anterior midgut of the insect [8], and it has been found in Dermaptera, Isoptera, Embiodea, and the larvae of Diptera [10]. 

The PM composed of proteins, proteoglycans and chitin, which together orchestrate the robust structure as well as the protective and semi-permeable functions of the matrix [11]. Several PM proteins have been described from sheep blow fly *Lucilia cuprina* [10], *Anopheles gambiae* [12], *Plutella xylostella* [13], *T.* *ni* [14,15], and *Holotrichia oblita* [16]. Based on the known characteristics, these proteins are classified into four groups. Group 3 proteins (named “peritrophins”) have been studied extensively, only extract with strong denaturants, and contain similar chitin binding domains (CBDs). Among them, three types of CBD (peritrophins A–C) have been identified based on the number and arrangement of conserved cysteine residues [8]. 

PM proteins contribute to the maintenance of the structural characteristics and biological functions of the PM by disulphide bonds, O-linked glycosylations or mutiple CBDs [8,17]. Any disruption of the integral peritrophic membrane may result in detrimental physiological effects to insects and even cause death, which makes this interesting in the expansion and research of new targets for pest control [11]. The study of the composition and biochemical properties of PM proteins are significant to elucidate the mechanism of the PM and the mechanism of interaction between the PM and virus or microorganisms. In this study, we identified and characterized a new PM protein HpCBP45 from *Holotrichia parallela* larvae by cDNA cloning. This protein possesses eight chitin-binding domains related to the peritrophin-A domain and its chitin-binding activity has been tested.

## 2. Results and Discussion

### 2.1. cDNA Cloning and Sequence Analysis of HpCBP45

**Figure 1:**
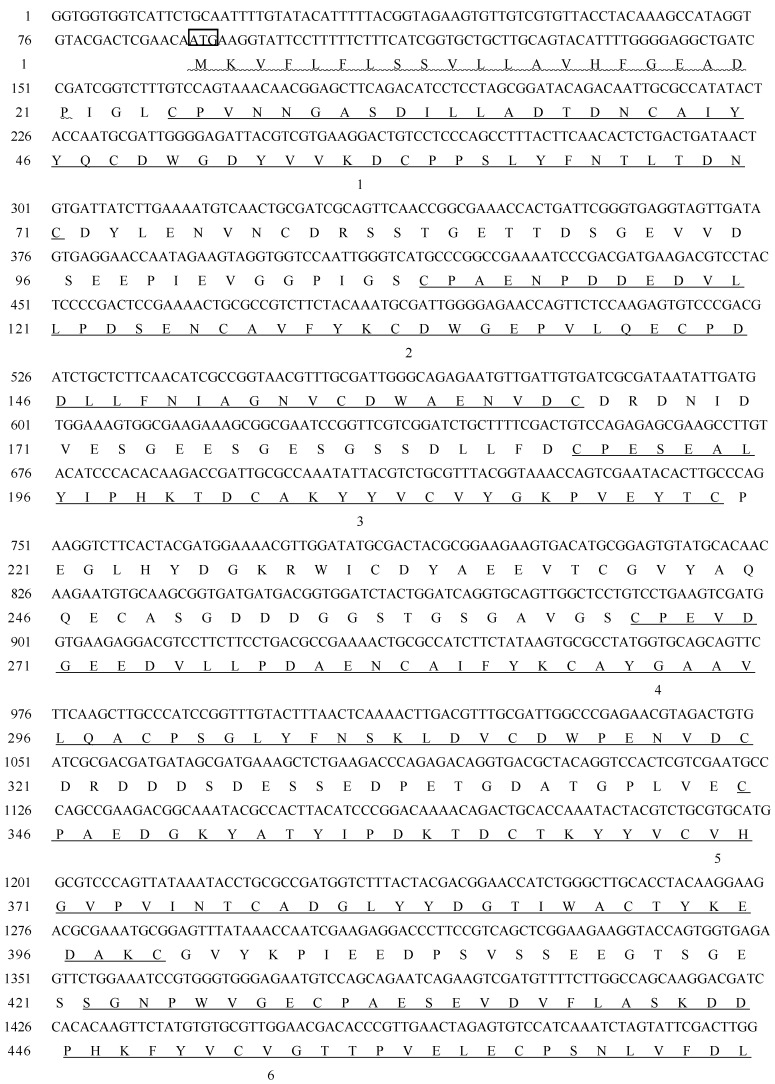

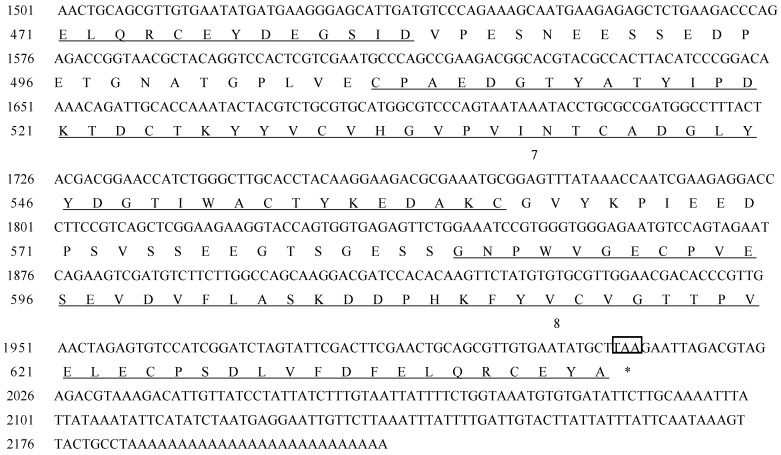
Nucleotide sequences of the cDNA for HpCBP45 and its deduced amino acid sequences. The translation initiation codon ATG and stop codon TAA are in box. The predicted signal peptide cleavage site is wavy underlined. eight chitin binding domains, peritrophin-A domains, are underlined and numbered from 1 to 8 from *N*- to *C*-terminus of the protein.

A *Holotrichia parallela* midgut cDNA expression library was screened with specific polyclonal antibody against PM proteins. To obtain positive clones, two rounds of screening were performed. Immunopositive clones were recovered and used for isolation of phagemid DNA which was sequenced, and a 2220 bp inserted cDNA fragment was identified. The nucleotide sequences analysis showed the cDNA fragment contained the start codon ATG and stop codon TAA, and a polyadenylation signal sequence AATAAA at the position of in front of the polyA, and was named *HpCBP45.* The open reading frame of *HpCBP45* is 1923bp, and encodes 641 amino acids. Nucleotide acid sequences was submitted to GenBank and its accession number is KC703970 (Figure 1). 

**Figure 2:**
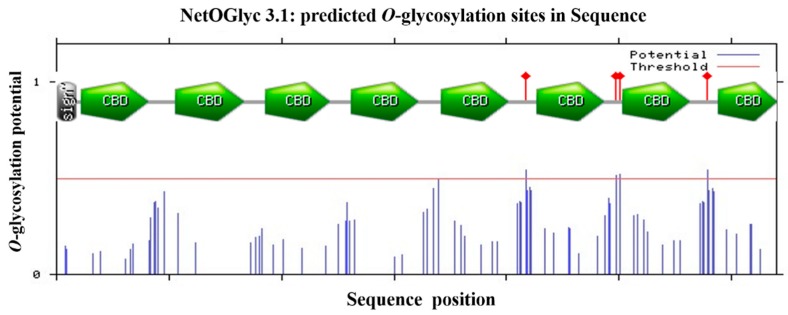
Schematic structures of the HpCBP45 and prediction analysis of glycosylation site. Functional domains were analyzed by Expasy prosite database. Residues with an -glycosylation potential exceeding the threshold level (red line) are expected to be glycosylated. Sign, signal peptide sequence; CBD, chitin-binding domain; Asn-Xaa-Ser/Thr sequons in the sequence output below are highlighted in blue.

It was predicted that there was a signal peptide of 21 amino acids at the N-terminal of the protein with a predicted molecular weight of 69.5 kDa. Analysis by the prosite database and by DNAMAN revealed that it consisted of eight tandem putative chitin binding domains with conserved sequence motifs CX_13–22_CX_5_CX_9_CX_12_CX_7_C, which are similar to the predicted chitin binding sequences from PM proteins of other species and belong to the peritrophin-A domains. The conversed cysteine residues are proposed to form three pairs of stable intradomain disulfide bonds cysteine residues which confer stability to the protein in the protease rich gut environment. The importance of disulfide bonds for the stability of PM proteins has been demonstrated in *Trichoplusia ni* PMs [16]. Prediction of potential glycosylation sites showed it contained four O-glycosylation and one potential *N*-glycosylation site located in 499, the gap between CBD2 and CBD7 (Figure 2). 

### 2.2. Phylogenetic Analysis of HpCBP45

**Figure 3:**
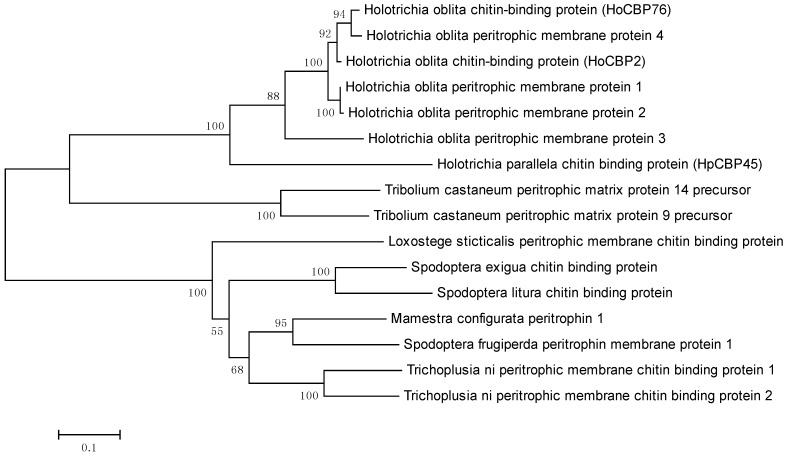
**Figure 3.** Phylogenetic tree based on amino acid sequence of HpCBP45 and other PM proteins.

Phylogenetic analysis of HpCBP45 with other PM proteins from different species was constructed MEGA5.0 Software (Figure 3). 

These PM proteins are from *Trichoplusia ni*, *Spodoptera frugiperda*, *Spodoptera litura*, *Mamestra configurata*, *Spodoptera exigua,* *Loxostege sticticalis*, *Holotrichia oblita*, *Tribolium castaneum*. The phylogenetic analysis showed that the amino acid sequence of HpCBP45 was closely related to those from other coleoptera PM proteins, and the highest degree of homology was with *Holotrichia oblita* peritrophic membrane proteins. 

### 2.3. Expression of Recombinant HpCBP45

**Figure 4:**
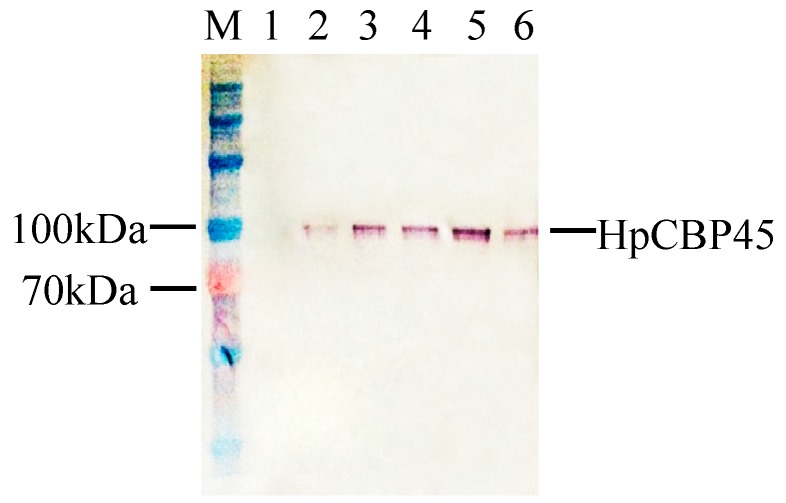
Western blot analysis of *HpCBP45* expression in recombinant yeast.

The results showed the recombinant protein reached a maximum accumulation in the medium at 72 h post-infection, while none appeared in the control. Western blot analysis showed a predominant band on the SDS-PAGE gel with an apparent molecular weight of 100 kDa, significantly higher than the predicted molecular weight of 69 kDa (Figure 4). It is presumed that the post-translational modifications and processing expressed in cells may attribute to the high molecular weight as many *O*-glycosylation sites appeared [18]. This phenomenon has been identified in other PM proteins.

M: Protein maker; 1: CK-, GS115 (pPIC9K) cells; 2–6, the expression of HpCBP45 in the GS115 (pPIC9K-HpCBP45) system for 12 h, 24 h, 48 h, 72 h, 96 h, respectively.

### 2.4. Chitin-Binding Activity of Recombinant HpCBP45

**Figure 5:**
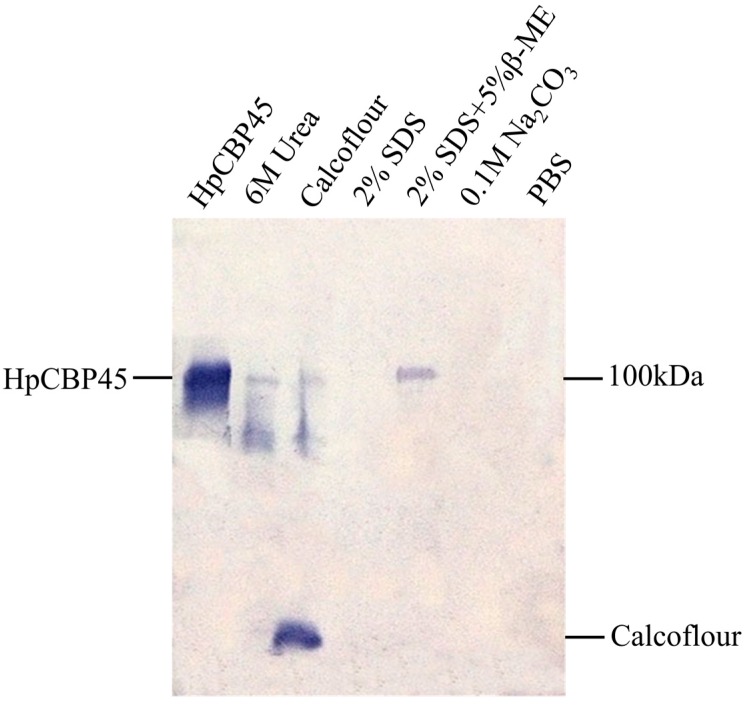
Western blot analysis for chitin binding activity of recombinant HpCBP45.

Subsequent chitin binding assays demonstrated that the recombinant HpCBP45 had chitin binding affinity (Figure 5). The HpCBP45 tightly bound to chitin and did not dissociate from the chitin following treatment with PBS, and 0.1 M Na_2_CO_3_. The proteins were only partially dissociated with 2% SDS in the presence of 5% β-mercaptoethanol. However, they were solubilized from the bound chitin by 2% SDS, 6 M urea or by 1% Calcofluor. Chitin binding activity of the peritrophic membrane protein plays an important role in maintaining the stability of the peritrophic membrane, This could explain its strong association with the PM and possibly its putative role in determining the porosity of this semi-permeable matrix [8,19].

HpCBP45: Culture media of recombinant HpCBP45 from GS115 (pPIC9K-HpCBP45) system. Recombinant HpCBP45 dissociated from the HpCBP45/chitin complex by incubation with 6 M urea, 1% Calcoflour, 2% SDS, 2% SDS + 5% β-ME, 0.1 M Na_2_CO_3_ were used for western blot.

### 2.5. HpCBP45 Transcriptional Analysis in Different Tissues

**Figure 6:**
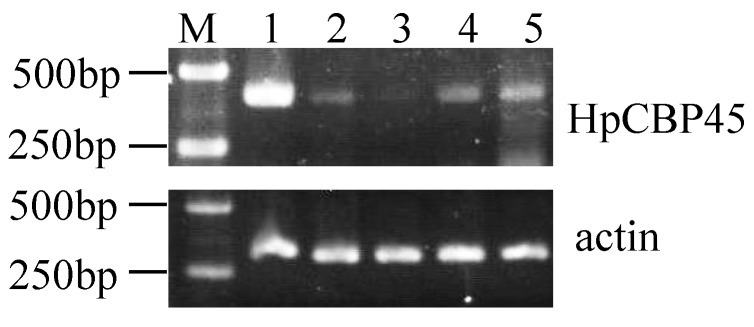
RT-PCR analysis of *HpCBP45* in different tissues.

The transcript levels of *HpCBP45* first examined by semi-quantitative RT-PCR using specific primers on RNA extracted from different tissues (Figure 6). *HpCBP45* gene had higher expression levels in the midgut, relatively low levels in head and fat body, and similar moderate levels in the moult and egg. 

Total RNA from 1: Midgut, 2: Exuviae, 3: Head, 4: Fat body and 5: Egg were used, and expression levels were normalized with housekeeping gene actin.

**Figure 7:**
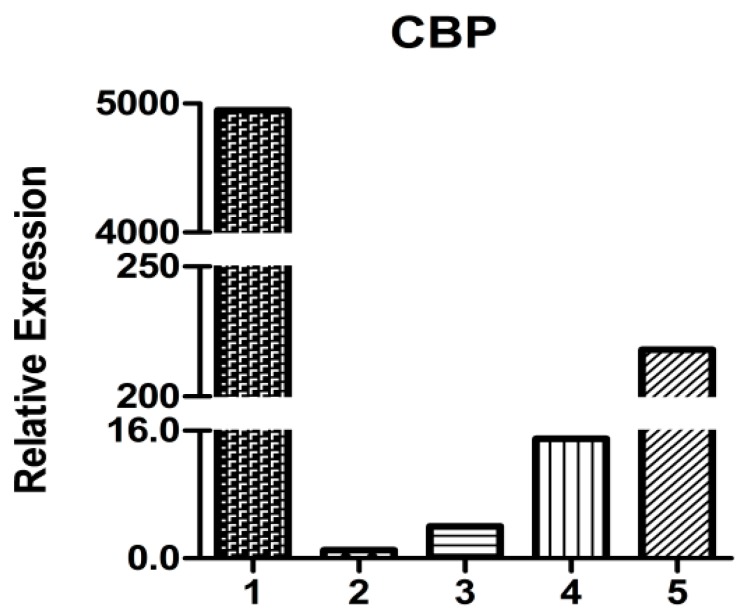
Relative expression of *HpCBP45* in different tissues.

As shown by qRT-PCR analysis (Figure 7), *HpCBP45* relative expression in the midgut is 20–5000-fold higher than the other tissues. In *H. parallela* larvae, the HpCBP45 was abundant in the midgut, suggesting that HpCBP45 was secrected by the whole midgut epithelium, which confirmed that the *H. parallela* PM belonged to the TypeⅠPM. The result was similar to the observations made in other Coleoptera insects. The *Diabrotica undecimpunctata* (Coleoptera: Chrysomelidae) PM was synthesized along the length of the midgut epithelium, the PM being secreted into the interstices between the microvilli of the brush border where it became organized into a coherent matrix and then moved apically along the microvilli to be shed from the tips of microvilli into the midgut lumen [20]. The high expression of *HpCBP45* gene in midgut and PM provides evidence for the structural and protective biological functions of the PM and may be involved in determining the ultrafilter character of the membrane.

Total RNA from 1: Midgut, 2: Head, 3: Exuviae, 4: Fat body and 5: Egg were used. and expression levels were normalized with housekeeping gene actin.

## 3. Experimental Section

### 3.1. Insect Larvae and PM Preparation

The *H. parallela* larvae were reared under laboratory conditions at 26 ± 1 °C, 60%–70% soil relative humidity. The PMs and various tissues were isolated from third instar larvae for analysis.

### 3.2. Cloning of H. Parallela Chitin Binding Protein cDNA and Sequence Analysis

PM proteins were precipitated with the addition of trichloroacetic acid (TCA) and the antiserum to PM proteins was generated by immunizing rabbit. Preimmune serum from the rabbit was collected [12]. A cDNA expression library of *H. parallela* midgut was screened by immunoscreening using antibodies against a collection of PM proteins. The screening procedure was according to the picoblue^TM^ immunoscreening Kit (Stratagene, La Jolla, CA, USA). From purified positive phages, the new PM protein cDNA clones were finally processed for in vivo excision to rescue the pBluescript phagemids following the ZAP-cDNA Gi-gapack cloning kit protocol (Stratagene). Plasmid DNA was isolated from the positive clone and subjected to sequencing. Sequence features of the cDNA was analyzed using the DNAMAN software and NCBI BLAST [21]. The signal peptide was predicted with signalP 4.0 and the prosite was analyzed with prosite database. The glycosylation was predicted with NetOglyc 3.1 server and NetNGlyc1.0 server [22] respectively. The phylogenetic tree was constructed by the neighbor-joining method of MEGA. 

### 3.3. Preparation of Expression Construct for Recombinant HpCBP45

Primers for the predicted *HpCBP45* (without the signal peptide sequence) was designed by primer primer 5.0, which had extra bases added to include *Eco*RI (N-terminal) and *Not*I (C-terminal) restriction sites: Forward, 5ꞌ-GGAATTCCTGCTTGCAGTACATTTTGGG-3' and Reverse, 5ꞌ-AAGGAAAAAAGCGGCCGCTTAATGATGATGATGATGATGATATTCACAACGCTGCAGTTCG-3ꞌ. The cDNA coding was subcloned into the expression vector pPIC9K, generating pPIC9K-HpCBP45, and transformed into the GS115 strain of *Pichia pastoris* using the electroporation method. The cells where then plated on minimal dextrosemedium (MD) agar (1.34% yeast nitrogen base, 0.00004% biotin, and 1% dextrose) a medium devoid of histidine where only the transformed cells can grow. To find a highly producing clone, over 60 colonies were grown in YPD (1% yeast extract, 2% peptone, 2% dextrose) containing different concentrations of geneticin. All expression constructs were sequenced to ensure that no errors had been introduced into the expressed polypeptides by the PCR process, and the construct had been correctly assembled.

### 3.4. Expression of Recombinant Chitin Binding Protein HpCBP45

Selected positive colonies were screened for expression of the recombinant protein for 72 h at 30 °C in YPD/G418 medium (3.0 mg/mL). The colonies were grown in 10 mL buffered glycerol-complex (BMGY) medium (1% yeast extract, 2% peptone, 100 mM potassium phosphate, pH 6.0, 1.34% YNB, 0.00004% biotin and 1% glycerol) at 30 °C for 24 h. Then cells were collected by centrifugation and resuspended in 15 mL buffered methanol-complex medium (BMMY) medium (same as BMGY but containing 1% methanol instead of 1% glycerol) and grown for 96 h to induce the production of recombinant protein. The supernatant of all the clones was detected with anti-His (C-term) primary antibodies followed by AP-linked goat anti mouse secondary Ig (IgG-AP) (BOSTER, Wuhan, China). 

### 3.5. Chitin-Binding Assay

The chitin-binding activity of HpCBP45 was analyzed using the chitin-binding assaying method described by Wang *et al.* [16]. The binding assay mixture contained 40 mg of regenerated chitin and 1 mL HpCBP45-containing cell culture medium, and HpCBP45 protein was allowed to bind to chitin at 4 °C in suspension overnight in the presence of 1 mM EDTA and 1mM phenylmethylsulfonyl fluoride. The regenerated chitin bound with HpCBP45 was washed three times with PBS, followed by centrifugations. Aliquots of the resulting chitin bound with HpCBP45 were incubated with 1% Calcoflour, 6 M urea, 2% SDS + 5% β-mercaptoethanol, PBS, 2% SDS and 0.1 M Na_2_CO_3_, respectively. After 1h incubation, the supernatants containing the HpCBP45 protein released from the chitin were collected and analyzed by SDS-PAGE.

### 3.6. Semi-Quantitative RT-PCR and Quantitative Real-Time RT-PCR

Semi-quantitative RT-PCR and quantitative real-time RT-PCR (qRT-PCR) was carried out to assess *HpCBP45* transcriptional levels. Total RNA was isolated from the midgut, exuviae, head, fat body and egg using the Purelink RNA Micro kit, according to the manufacturer’s instructions (Invitrogen, Life Technologies, Carlsbad, CA, USA). First-strand cDNA samples were generated from 2 μg of total RNA, using the RT-PCR kit (Promega, Madison, Wisconsin, USA). The housekeeping gene actin was used to optimize cDNA concentration for PCR. The mRNA abundance of *HpCBP45* gene in different tissues was estimated by semi-quantitative RT-PCR. The following primers were used: 5'-AAACCACTGATTCGGGTGAG-3' and 5'-TCTTCATCGTCGGGATTTTC-3' (97 bp) for *HpCBP45*, 5ꞌ-ATGTTG CCATCCAAGCTGTA-3' and 5'-CCAAACGCAAAATAGCATGA-3' for actin (138 bp).

qRT-PCR was conducted using the Takara SYBR *Premix Ex Taq*^TM^ Ⅱreagent on an iQ™5 Optical System (Bio-Rad, Hercules, CA, USA). Accumulation of amplified product as detected by increased SYBR Green I fluorescence was measured in real time. Expression levels were all normalized using the expression levels of a non-differentially expressed actin gene for comparison. PCR conditions were: 40 Cycles of 95 °C for 5 s, and 65 °C for 30 s. After melting curve analysis, the relative quantities of *HpCBP45* transcripts were assessed using the 2^−∆∆Ct^ method [23].

## 4. Conclusions

In this study, a full-length cDNA gene *HpCBP45* obtained by screening the cDNA expression library of *Holotrichia parallela* larvae midgut was characterized based on clone sequencing and expression analysis. The recombinant protein HpCBP45 belonged to class 3 PM proteins and exhibited its capability to bind chitin. The chitin-bound protein could only be released from the chitin by 1% Calcofluor and 6 M urea. Sequence alignment and phylogenetic tree showed that HpCBP45 was closely related to TypeⅠPM protein from larval *Holotrichia oblita*, while qRT-PCR analysis demonstrated that the relative expression of *HpCBP45* gene was primarily synthesized in the midgut, which confirmed that the *H. parallela* PM belonged to the Type I PM. The study of the composition and biochemical properties of PM protein has significance to elucidate the mechanism of formation of the peritrophic membrane and the mechanism of interaction between the peritrophic membrane and plants or pathogenic microorganisms. This could provide a theoretical reference for the research of pest targets.
